# The Emerging Roles of Axonemal Glutamylation in Regulation of Cilia Architecture and Functions

**DOI:** 10.3389/fcell.2021.622302

**Published:** 2021-03-04

**Authors:** Wen-Ting Yang, Shi-Rong Hong, Kai He, Kun Ling, Kritika Shaiv, JingHua Hu, Yu-Chun Lin

**Affiliations:** ^1^Institute of Molecular Medicine, National Tsing Hua University, HsinChu City, Taiwan; ^2^Department of Biochemistry and Molecular Biology, Mayo Clinic, Rochester, MN, United States; ^3^Division of Nephrology and Hypertension, Mayo Clinic, Rochester, MN, United States; ^4^Mayo Clinic Robert and Arlene Kogod Center on Aging, Mayo Clinic, Rochester, MN, United States; ^5^Department of Medical Science, National Tsing Hua University, HsinChu City, Taiwan

**Keywords:** primary cilia, motile cilia, tubulin glutamylation, ciliopathies, chemically inducible dimerization

## Abstract

Cilia, which either generate coordinated motion or sense environmental cues and transmit corresponding signals to the cell body, are highly conserved hair-like structures that protrude from the cell surface among diverse species. Disruption of ciliary functions leads to numerous human disorders, collectively referred to as ciliopathies. Cilia are mechanically supported by axonemes, which are composed of microtubule doublets. It has been recognized for several decades that tubulins in axonemes undergo glutamylation, a post-translational polymodification, that conjugates glutamic acid chains onto the C-terminal tail of tubulins. However, the physiological roles of axonemal glutamylation were not uncovered until recently. This review will focus on how cells modulate glutamylation on ciliary axonemes and how axonemal glutamylation regulates cilia architecture and functions, as well as its physiological importance in human health. We will also discuss the conventional and emerging new strategies used to manipulate glutamylation in cilia.

## Cilia and Ciliopathies

### The Architecture of Cilia

**FIGURE 1:**
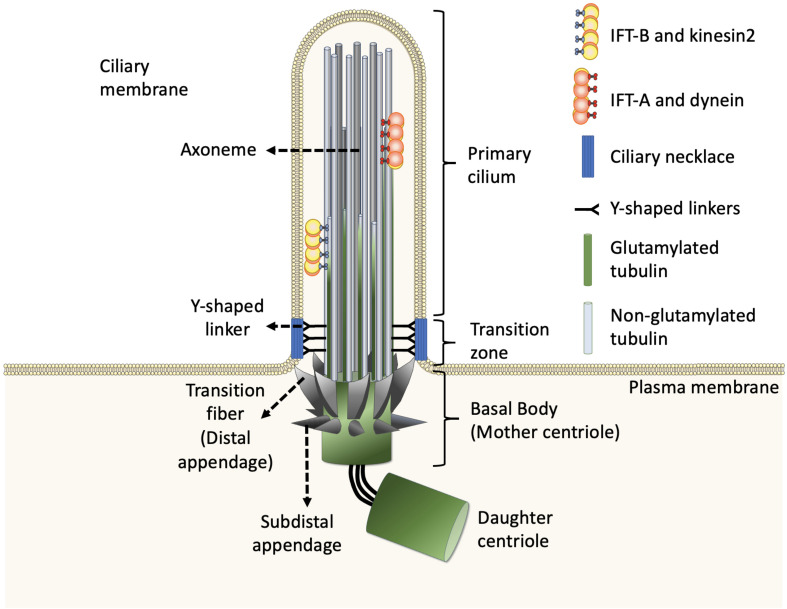
Structure of the primary cilium. The core primary cilium is a solitary organelle which can be segregated into upper and lower half. The upper half is comprised of axonemes which are typically formed by nine peripheral microtubule doublets arranged radially, enclosed within ciliary membrane. The axoneme comprises doublets built by A-tubule (gray) and glutamylated B-tubule (green) at the proximal part of cilia. The doublets transit to non-glutamylated A-tubule singlet at the middle and distal parts of cilia. The ciliary base further forms two sub-regions namely, transition zone and transition fibers, respectively. The transition zone lies between axoneme and transition fiber. The transition fibers emerge from the basal body in a spoked fashion, serving as a link between basal body and ciliary membrane. The Y-shaped linkers reside in transition zone and connect axoneme to the ciliary necklace. Dynein moves IFT-A complex and its cargo toward the cell body whereas, kinesin2 moves IFT-B complex and its cargo toward the ciliary tip, along the axoneme.

**FIGURE 2:**
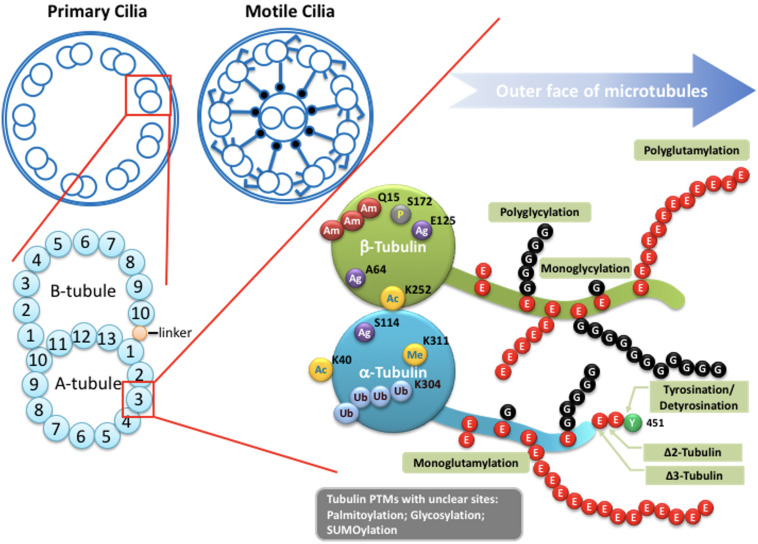
Potential tubulin PTMs along the axoneme. Upper left: Schematic drawings of cross-section of the axoneme in primary cilia or motile cilia. Lower left: the doublets of the axoneme are hollow tubes composed of 13 (A-tubule) or 10 (B-tubule) protofilaments. Lower right: the wall of each protofilament is assembled from the globular domain of α-tubulin–β-tubulin dimers, with unstructured carboxy-terminal segment facing outer surface of the axoneme. PTMs with known molecular location on the globular part of tubulins include acetylation, phosphorylation, polyamination, ubiquitination, arginylation, and methylation. Globular PTMs usually occur at fixed molecular locations. Some PTMs preferentially choose certain tubulin subtypes, such as K40 acetylation specifically occurs on α-tubulins at the luminal surface of microtubules, while the Q15 polyamination appears to present on β-tubulins. Detyrosination/tyrosination and D2/D3-tubulin modifications are limited to the very end of the carboxy-terminal tail of a-tubulins. For tyrosination, a modified nitrotyrosine (NO_2_Tyr) could be incorporated into the extreme carboxyl terminus of α-tubulin via the same tubulin tyrosination enzyme (Eiserich et al., 1999). (Poly)glycylation and (poly)glutamylation are found along the glutamate-rich carboxy-terminal tails of both α- and β-tubulins. Several other PTMs, including palmitoylation, glycosylation and SUMOylation, have been identified on tubulins, but with little knowledge regarding to molecular location and physiological importance. Ac: Acetylation. Am: Polyamination. Ag: Agination. Me: methylation. P: Phosphorylation. Ub: Polyubiquitination. E: Glutamate. G: Glycine.

The cilium is a hair-like organelle ubiquitously found on the surface of eukaryotic cells, each of which has a core formed by a microtubule-based axoneme and a basal body (transformed from the mother centriole) that anchors the cilium (Figure 1). Functionally, there are two different types of cilia: motile cilia (or termed as flagella in some eukaryotic cells) or non-motile cilia (or primary cilia) (Figure 2). In general, the shaft of the cilium is supported by a ring of nine outer microtubule doublets, with an extra central pair of doublets in the motile cilium (termed 9 + 2 arrangement), but not in the primary cilium (termed 9 + 0 arrangement) (Figure 2). Other than the central pair of microtubule doublets, motile cilia also possess unique structures such as dynein arms, radial spokes, and nexin-dynein regulatory complex (N-DRC), which attach to outer doublets and act together to produce ATP-driven beating or waving motion of motile cilia. With this kinetic capability, motile cilia can propel the movement of the ciliated cells/organisms, or generate fluid flow on the surface of the ciliated cells. In contrast to the force-generating motile cilium, the primary cilium is recognized as a sensory organelle, which acts like a cell’s antenna to recognize, integrate, and transform extracellular cues into internal signal transduction cascades that allows the cell to perceive and respond properly to its microenvironment (Goetz and Anderson, 2010; Patel and Honoré, 2010; Louvi and Grove, 2011).

In general, the structures of both motile cilia and primary cilia are highly conserved during evolution. Evidence from electron microscopy and super-resolution microscopy shows that each cilium can be divided into distinct and conserved domains: a microtubule-based axoneme as the ciliary shaft, a basal body supporting the protrusion of the axoneme, and microdomains on the proximal end of the axoneme including pinwheel-like transition fibers that connect the distal end of the basal body to the ciliary membrane, the transition zone with Y-shaped linkers connecting the axoneme to a specialized membrane domain known as the ciliary necklace (Figure 1; Reiter et al., 2012; Gonçalves and Pelletier, 2017).

### Ciliopathies

Primary cilium acts as a central hub for a wide spectrum of signaling pathways required for embryonic development and tissue homeostasis, such as hedgehog (Hh), canonical and non-canonical Wingless (WNT), transforming growth factor-β (TGF-β), platelet-derived growth factor receptor (PDGFR), and various G protein-coupled receptor (GPCR) signalings (Goetz and Anderson, 2010; Nishimura et al., 2019). With rapid advancements in next-generation sequencing and its application in human genetics, at least 187 causal loci have been cloned in 35 rare human disorders, such as polycystic kidney disease (PKD), Bardet-Biedl syndrome, Joubert syndrome, and Meckel-Gruber syndrome, which are now collectively termed as cilia-related diseases, or ciliopathies (Badano et al., 2006; Adams et al., 2008; Reiter and Leroux, 2017). Of note, since all ciliopathies are rare genetic diseases, a significant share of causal loci have not been cloned yet due to their extremely low incidence. Consistent with the fact that cilia are ubiquitously present in the human body, ciliopathies usually occur as syndromic disorders that share common manifestations (such as brain anomalies, retinal degeneration, kidney and liver dysfunction, skeletal abnormalities, obesity/diabetes, infertility, and situs inversus) (Hildebrandt et al., 2011). Despite the importance of cilia in both cell biology and human health, many central questions in the context of cilia, especially primary cilia; including how cilia in different cell types are modified to execute sensory functions, how cilia signalings convert into specific cellular behaviors, and most importantly, the molecular function of most identified ciliopathy proteins, remain poorly understood.

### Intraflagellar Transport Builds and Maintains All Cilia

Inside the cilium, the axoneme is closely covered by the ciliary membrane and thus possesses limited cytosolic space. Extensive electron microscopy studies of cilia across various organisms in the last few decades conclude that there is no presence of the ribosome inside cilia. Thus, all ciliogenic proteins required for cilia biogenesis, maintenance, and function need to be synthesized in the cytoplasm and then sorted into the cilium via intracellular trafficking routes. All cilia and eukaryotic flagella are built and maintained by phylogenetically conserved intraflagellar transport (IFT) machinery (Rosenbaum and Witman, 2002). IFT consists of bidirectional movement along the axoneme (Figure 1; Satir and Christensen, 2007; Pedersen and Rosenbaum, 2008; Hao et al., 2009; Pedersen and Christensen, 2012; Lechtreck, 2015; Prevo et al., 2017). Anterograde movement of particles away from the ciliary base is mediated by kinesin-2 motor proteins, whereas retrograde movement away from the ciliary tip is powered by cytoplasmic dynein motor proteins. The particles transported by IFT are composed of at least 20 protein subunits, that form two distinct complexes known as the IFT-A complex and IFT-B complex (Hao et al., 2009; Pedersen and Christensen, 2012; Broekhuis et al., 2013; Lechtreck, 2015; Prevo et al., 2017). In general, kinesin-2 and cytoplasmic dynein bind to the IFT-B complex and IFT-A complex, respectively. Depletion or mutation of most of the IFT components affects cilia biogenesis and ciliary signalings in almost all ciliated organisms, revealing that the role of IFT machinery is fundamentally conserved (Hao et al., 2009; Pedersen and Christensen, 2012; Broekhuis et al., 2013; Lechtreck, 2015; Prevo et al., 2017).

### Tubulin Post-translational Modifications of the Ciliary Axoneme

The axoneme serves as the skeleton of the primary cilium, giving support to its structure and, most importantly, providing a track for IFT-dependent movement (Figure 1; Singla and Reiter, 2006). Like other larger structures formed by microtubules, such as the mitotic spindle, the cilium consists of microtubules assembled from heterodimers of α- and β-tubulin into long, and polarized hollow polymers, including the ciliary tip (a plus-end) with exposed β-tubulin and the proximal end of the basal body (a minus-end) with exposed α-tubulin. The doublets of the axoneme are formed by a full-circle A-tubule (with 13 protofilaments) attached by an incomplete B-tubule (with 10 protofilaments) (Figure 2; Ichikawa et al., 2017; Ma et al., 2019). To adapt to a large diversity of functions as well as to generate spatiatomporal specialized identities, the microtubules could be comprised of different tubulin isotypes or undergo many highly conserved post-translational modifications (PTMs), including acetylation, detyrosination, Δ2/Δ3 modification, glutamylation, glycylation, and phosphorylation, as well as poorly studied ones including palmitoylation, glycosylation, arginylation, methylation, and SUMOylation, which are collectively referred to as the “tubulin code” (Gaertig and Wloga, 2008; Wloga and Gaertig, 2010; Janke and Bulinski, 2011; Konno et al., 2012; Janke, 2014). For tubulin PTMs with known modifications sites, many of them occur in the C-terminal region of tubulins that protrudes from the outer surface of microtubules (Figure 2; L’Hernault and Rosenbaum, 1985; LeDizet and Piperno, 1987; Janke and Bulinski, 2011; Soppina et al., 2012; Gadadhar et al., 2017a). Evidence from various organisms and mammalian cell types indicates that tubulin PTMs usually are present on long-lived and stabilized subsets of microtubule polymers. However, the debate about whether PTMs contribute to microtubule stability or, in contrast, whether only older polymers gain access to PTM enzymes has so far been inconclusive. The molecular localization and unstructured property of the C-terminal region of tubulins naturally provides a flexible interface that potentially influences the association between microtubules and microtubule-binding proteins such as, microtubule-associated proteins (MAPs), kinesin, and dynein motors that transport IFT machinery. As MAPs regulate the properties of microtubules and IFT builds and maintains all cilia, it is widely believed that axonemal PTMs are important for regulation of cilia structure and functions (Gaertig and Wloga, 2008; Janke and Bulinski, 2011). In this review, we specifically focus on the emerging role of microtubule glutamylation, one of the most abundant PTMs in ciliary axonemes, which results in the covalent conjugation of one (through the γ-carboxyl group) or more (through the subsequent α-carboxyl groups) glutamate residues onto the C-terminal tail of axonemal tubulins (Gaertig and Wloga, 2008). We describe how cells modulate glutamylation on ciliary axonemes and how axonemal glutamylation regulates cilia architecture and functions.

## Modulation of Glutamylation on Ciliary Axonemes

### Tubulin Glutamylation-Catalyzing Enzymes

**FIGURE 3:**
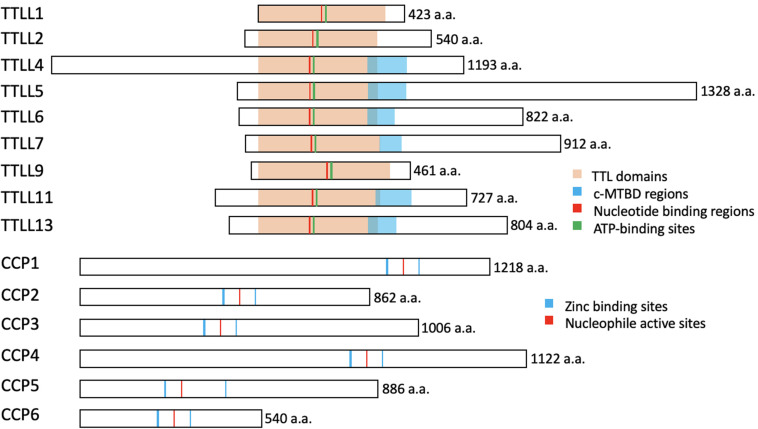
The conserved structures of the TTLL glutamylases and the CCP deglutamylases in mice. The illustrations represent the murine TTLL proteins that function as glutamylases (upper) and the murine CCP proteins that function as deglutamylases (lower). The enzymatic core TTL domains (orange), ATP-binding sites (green), and the nucleotide-binding regions (red) are highly conserved, whereas the c-MTBD regions (blue) are conserved in the autonomous TTLL proteins (TTLL4, 5, 6, 7, 11, and 13) for their interactions with microtubules. Note that TTLL1, 2, and 9 lack the c-MTBD region and function as the catalytic subunits in protein complexes. The CCP proteins belong to the M14 metallocarboxypeptidase family and have highly conserved zinc binding sites (blue) and a nucleophile active site (red). These illustrations are based on sequences obtained from UniProt (www.uniprot.org): TTLL1, Q91V51; TTLL2, A4Q9E4; TTLL4, Q80UG8; TTLL5, Q8CHB8; TTLL6, A4Q9E8; TTLL7, A4Q9F0; TTLL9, A2APC3; TTLL11, A4Q9F4; TTLL13, A4Q9F6; CCP1, Q641K1; CCP2, Q8CDK2; CCP3, Q8CDP0; CCP4, Q09M05; CCP5, Q09M02; CCP6, and Q09LZ8.

The enzymes required for tubulin glutamylation belong to the tubulin tyrosine ligase-like (TTLL) family, of which the members share a conserved tubulin tyrosine ligase (TTL) core and a cationic microtubule-binding domain (c-MTBD) (Figure 3; Van Dijk et al., 2007; Garnham et al., 2015). TTLLs adopt a tripartite strategy for substrate recognition: TTLLs attach to the ionic C-terminal tail of tubulin via their TTL core, bind to microtubules with the c-MTBD, and eventually position themselves for subsequent modifications (Garnham et al., 2015). There are 13 TTLL family members in mammals. Nine of these are characterized as glutamylases, including TTLL1, 2, 4, 5, 6, 7, 9, 11, and 13, whereas the rest act as glycylases or tyrosinases (Table 1; Van Dijk et al., 2007). According to the architectural differences in their core domains and affinities for substrate binding, TTLLs exhibit enzymatic preferences for either α- or β-tubulin and specificities with respect to the type of glutamylation reactions, such as chain initiation or elongation (Table 1; Natarajan et al., 2017; Mahalingan et al., 2020). A recent work revealed that the corresponding residues Q180 and H362 of TTLL6 determine the reaction specificity for elongation instead of initiation. The substitution with Arg at the position Q180 allows the formation of hydrogen bond with acceptor glutamate, which thereby favors the γ-linked glutamylation. H362, on the other hand, stabilizes the intermediate with van der Waals for the initiation reaction. The substitution with Ile does not only disrupt this interaction but also neutralize the carboxylate of the acceptor glutamate as what other elongases do (Mahalingan et al., 2020). The flexibility of the catalytic core in mammalian TTLL7 allows microtubule docking for execution of both chain initiation and elongation reactions on β-tubulin, whereas others function only as initiates or elongases (Table 1; Van Dijk et al., 2007; Mukai et al., 2009; Garnham et al., 2015; Natarajan et al., 2017; Mahalingan et al., 2020). Some TTLLs function autonomously, whereas others may require the assistance of associated activators for their functions, such as TTLL1, 2, and 9 (Figure 3 and Table 1; Janke et al., 2005; Van Dijk et al., 2007).

**TABLE 1:** Members of the TTLL family that act as glutamylases.

Enzyme and Homologs	PTM	Substrate Preference	Reaction Specificity	Autonomy	Subcellular/Ciliary Distribution	References
TTLL1 Ttll1p (*Tetrahymena thermophila*) LmTTLL1 (*Leishmania major*)	Glutamylation	α-Tubulin Non-Tubulin Protein(s): Klf4	Initiation	One subunit of a complex	Basal Bodies Contractile Vacuole Pores Oral Deep Fibers Cell Body (excluded from the nucleus)	Janke et al., 2005; Van Dijk et al., 2007; Wloga et al., 2008; Ikegami et al., 2010; Vogel et al., 2010; Pathak et al., 2011; Ye et al., 2018; Lan et al., 2020
*TTLL2	Glutamylation	Unknown	Unknown	One subunit of a complex	Unknown	Van Dijk et al., 2007
TTLL4 LmTTLL4A (*Leishmania major*) LmTTLL4B (*Leishmania major*) LmTTLL4C (*Leishmania major*)	Glutamylation	β-Tubulin Non-Tubulin Protein(s): PELP1, Mad2, NAPs (NAP1 an NAP2)	Initiation	Autonomous	Basal Bodies Cilia Cell Body Nucleus Mitochondria Mitotic Spindles Mid-Bodies	Van Dijk et al., 2007; Kashiwaya et al., 2010; Kimura et al., 2010; Lacroix et al., 2010; Rogowski et al., 2010; O’Hagan et al., 2011; Pathak et al., 2011; Ye et al., 2014; Casanova et al., 2015; Chawla et al., 2016; Ijaz et al., 2017; Kimura et al., 2018; Ye et al., 2018; Mahalingan et al., 2020
TTLL5	Glutamylation	α-Tubulin Non-Tubulin Protein(s): RGPR	Initiation	Autonomous	Basal Bodies Cilia	Van Dijk et al., 2007; Ghosh-Roy et al., 2012; Lee et al., 2013; Chawla et al., 2016; Sun et al., 2016; Devambez et al., 2017; Bompard et al., 2018; He et al., 2018
TTLL6 Ttll6Ap (*Tetrahymena thermophila*) LmTTLL6A (*Leishmania major*) LmTTLL6B (*Leishmania major*)	Glutamylation	β-Tubulin α-Tubulin (under the overexpression condition in HeLa cell) Non-Tubulin Protein(s): Mad2	Elongation §LmTTLL6B: specific to initiation	Autonomous	Basal bodies Cilia B-tubules of the outer doublets Cell Body (excluded from the nucleus) §Also found in the nucleus of low ploidy megakaryocytes §Also found in tau missorting dendrites §LmTTLL6B: Also found as an additional intense dot at the posterior end of L. major	Janke et al., 2005; Van Dijk et al., 2007; Wloga et al., 2008; Lacroix et al., 2010; Suryavanshi et al., 2010; Pathak et al., 2011; Grau et al., 2013; Zempel et al., 2013; Casanova et al., 2015; Ye et al., 2014; He et al., 2018; Mahalingan et al., 2020
TTLL7	Glutamylation	β-Tubulin	Initiation and Elongation	Autonomous	Basal Bodies Cilia	Ikegami et al., 2006; Van Dijk et al., 2007; Mukai et al., 2009; Pathak et al., 2011; He et al., 2018
TTLL9 Ttll9p (*Tetrahymena thermophila*) tpg1 (*Chlamydomonas reinhardtii*) LmTTLL9 (*Leishmania major*)	Glutamylation	α-Tubulin	Elongation	One subunit of a complex	Basal Bodies Cilia Cell Body (excluded from the nucleus)	Van Dijk et al., 2007; Wloga et al., 2008; Ghosh-Roy et al., 2012; Kubo et al., 2014; Casanova et al., 2015
TTLL11	Glutamylation	α-Tubulin	Elongation	Autonomous	Basal Bodies Cell Body Axon Dendrites Cilia §Exists as puncta in the sensory neurons	Van Dijk et al., 2007; Lacroix et al., 2010; Chawla et al., 2016; O’Hagan et al., 2017
TTLL13	Glutamylation	α-Tubulin	Elongation	Autonomous	Unknown	Van Dijk et al., 2007; Bompard et al., 2018

*^∗^TTLL2 is believed to be a glutamylase based on its homology to other family members; however, further research is required for its definitive characterization.*

In addition to their different enzymatic preferences, TTLL glutamylases also exhibit distinct subcellular distributions. Although there is no antibody that is specific for a particular TTLL subtype, fluorescent protein–labeling or tag-labeling experiments have demonstrated that TTLL1, 9, and 11 are mainly localized to the basal body, whereas TTLL4, 5, 6, and 7 are localized to the basal body, and at the cilia shaft (Table 1; Van Dijk et al., 2007; He et al., 2018). Therefore, it is likely that axonemal glutamylation is regulated by these cilium-preferring TTLL proteins. Indeed, hypoglutamylation of axonemal microtubules induced by the depletion of TTLL4, 5, or 6 in primary cilia or motile cilia has suggested their key roles in regulating ciliary glutamylation. TTLL1, 9, and 11 in the basal body are also involved in axonemal glutamylation because of the critical relationship between basal body and cilia (Table 2; Wloga et al., 2008; Ikegami et al., 2010; Suryavanshi et al., 2010; Vogel et al., 2010; O’Hagan et al., 2011, 2017; Lee et al., 2012, 2013; Grau et al., 2013; Kubo et al., 2014; He et al., 2018; Kimura et al., 2018; Ki et al., 2020). Moreover, the localization of TTLL7 in cilia of transfected MDCK cells also implies that it has a role in ciliary glutamylation, although more studies are required to confirm this (Van Dijk et al., 2007).

**TABLE 2:** *In vivo* impacts of glutamylation alternation on cilia/flagellar.

Species	Change of Glutamylation	Cilia-related Phenotypes	Note	References
Human	Hyperglutamylation	N/A	N/A	N/A
	Hypoglutamylation	Joubert syndrome (JBTS15)	CEP41 mutation reduces ciliary entry of TTLL6	Lee et al., 2012
		Joubert syndrome (JBTS30)	ARMC9 and TOGARAM1 coordinate axoneme polyglycylation and polyglutamylation	Latour et al., 2020
		Retinal degeneration	TTLL5 homozygous mutation	Sergouniotis et al., 2014
Mouse	Hyperglutamylation	No primary cilia-associated phenotypes	*Ccp2* KO mice	Tort et al., 2014
		No primary cilia-associated phenotypes	*Ccp3* KO mice	Tort et al., 2014
		No primary cilia-associated phenotypes	*Ccp5* KO mice	Xia et al., 2016; Wu et al., 2017
		Infertile, no mature sperm	*Ccp5* KO mice	Wu et al., 2017; Giordano et al., 2019
		Shortening of connecting cilia; retinal degeneration	*Ttll3* KO mice lack glycylation in photoreceptors, which results in and hyperglutamylation	Bosch Grau et al., 2017
		Shortening of connecting cilia; retinal degeneration, abnormal sperm	*Ccp1*mutant mice	Mullen et al., 1976; Bosch Grau et al., 2017
	Hypoglutamylation	Primary ciliary dyskinesia (PCD)-like phenotypes and infertility in males	*Ttll1* deficiency	Ikegami et al., 2010; Vogel et al., 2010
		Reduce ependymal cilia beating frequency	*Ttll6* deficiency	Grau et al., 2013
		Aberrant sperm flagellar beating; shortened axoneme	*Ttll9* deficiency	Konno et al., 2016
		Infertile, defective sperm structure and motility	*Ttll5* KO mice	Lee et al., 2013
		Loss of tubulin glutamylation, infertile, abnormal sperm flagella	*ROSA22 mice that lack* PGs1, a non-catalytic subunit that associates with TTLL1	Campbell et al., 2002; Ikegami et al., 2007; Lee et al., 2013
**Zebrafish**	Hyperglutamylation	Axis curvature, hydrocephalus, pronephric cysts, and disrupts cilia motility	*Ccp1* or *Ccp5* depletion	Lyons et al., 2013; Pathak et al., 2014
		Shortening and loss of axonemes	*Ttll3* depletion	Wloga et al., 2009
	Hypoglutamylation	Mild structure and motility defects	*Ttll6* depletion	Pathak et al., 2011
*C. elegans*	Hyperglutamylation	Defective doublet structure; dysregulated ciliary kinesin motility; defective extracellular vesicles release	*ccpp-1* deficiency	O’Hagan et al., 2011, 2017
	Hypoglutamylation	Dysregulated kinesin motility; stabilized sealing between A- and B-tubules; defective extracellular vesicles release.	TTLL-11 deficiency	O’Hagan et al., 2017
		Reduced IFT along the axoneme upon starvation	TTLL-4 deficiency	Kimura et al., 2018
*Tetrahymena thermophila*	Hyperglutamylation	Shorter axoneme with normal structure; Paclitaxel resistance	Disruption of glycylase TTLL3 reduces glycylation but increases glutamylation	Wloga et al., 2009
		Paralyzed cilia and disrupted dynein-regulated motility	TTLL6 overexpression	Janke et al., 2005; Suryavanshi et al., 2010
		Destabilized axonemal microtubules	TTLL6 overexpression	Wloga et al., 2010
	Hypoglutamylation	Shorter cilia lack the central pair	βDDDE_440_ mutation of β-tubulin prevents glutamylation and glycylation.	Thazhath et al., 2002
		Slow maturation of basal bodies; Defective cilia functions	TTLL1 and TTLL9 deficiencies	Wloga et al., 2008
		Defective cilia motility caused by compromised sliding doublet microtubules by inner dynein arms	TTLL6 deficiency	Suryavanshi et al., 2010
		Basal bodies destabilize against ciliary beating force	TTLL1; TTLL9 double knockout cells	Bayless et al., 2016
C*hlamydomonas*	Hyperglutamylation	N/A	N/A	N/A
	Hypoglutamylation	Reduced flagellar motility but normal axonemal structure	Tbg1 (TTLL9) deficiency	Kubo et al., 2012, 2010
		Stable flagellar	Tbg1 (TTLL9) deficiency	Lin et al., 2015
		Stabilizes axonemal microtubules, decelerating axonemal disassembly.	Tbg1 (TTLL9) deficiency	Kubo et al., 2015

How cells glutamylate axonemes via TTLLs are carried out by two mechanisms: the recruitment of TTLL family members to the cilia and the activation of its enzyme activity. The axonemal glutamylation of sensory cilia in *Caenorhabditis elegans* is up-regulated in response to various environmental stimuli including heat, cold, high osmolarity, and starvation (Kimura et al., 2018). This is achieved by p38 MAPK–mediated TTLL4 activation, perhaps owing to the phosphorylation on Thr446 of TTLL4 (Kimura et al., 2018). The glutamylation of axonemal microtubules in primary cilia also relies on the spatial restriction of the corresponding TTLLs through their interacting proteins. During ciliogenesis in human retinal pigment epithelium (RPE) cells, ARL13B (ADP-ribosylation factor-like protein 13B) and RAB11/FIP5 (RAB11 family interacting protein1)-positive vesicles coordinately promote the transport of TTLL5- and TTLL6-containing vesicles to the ciliary base, which results in an increase in general polyglutamylation with long side chains in the cilia (He et al., 2018). In another population of ciliated cells, human umbilical vein endothelial cells, the centrosome protein CEP41 is able to regulate the level of glutamylation of the axonemal microtubules by controlling the ciliary entry of TTLL6. This CEP41/TTLL6-mediated glutamylation further promotes deciliation and the up-regulation of pro-angiogenesis factors in response to shear stress (Lee et al., 2012; Ki et al., 2020). The protist homolog of TTLL9 in *Chlamydomonas*, tpg1, forms a complex with a flagella-associated protein, FAP234, which mainly functions to stabilize and deliver TTLL9 into the flagella matrix via IFT trains (Kubo et al., 2014). Moreover, the centriole, cilia, and spindle-associated protein (CSAP) can form complexes with TTLL5 and other autonomously active TTLLs, including TTLL4, 6, 7, 11, and 13. CSAP and the binding TTLL5 reciprocally regulate one another, which not only promotes their localization to axonemal microtubules but also enhances tubulin glutamylation (Bompard et al., 2018). Whether these TTLL-interacting proteins confer the distinct distributions of each TTLL members in different model organisms merit comprehensive scrutiny.

### Tubulin Deglutamylation-Catalyzing Enzymes

As for the reverse modification, the tubulin deglutamylation function of cytosolic carboxypeptidases (CCPs) such as CCP1, 2, 3, 4, 5, and 6, has been explored in last two decades. CCPs belong to the M14 metallocarboxypeptidase family and have a conserved structure with a β-sheet–rich prodomain followed by the catalytic carboxypeptidase (CP) domain, which contains zinc-binding sites and an active nucleophile site (Figure 3; Kalinina et al., 2007; Berezniuk et al., 2012; Otero et al., 2012). The conserved Arg residue in the catalytic pocket and the neighboring basic residues confine the substrate preferences to acidic amino acids (Tort et al., 2014). A long polyglutamate side chain or nearby acidic residues in tubulin may increase the localized acidity and thus enhance the catalysis activity of the CCPs (Wu et al., 2015).

In addition, CCPs have enzymatic preference for the removal of branched glutamic acids or long polyglutamate side chains (Tort et al., 2014; Wu et al., 2015, 2017). Several previous studies suggest that CCP5 possesses a catalytic preference for the γ-carboxyl-linked glutamate, while others, CCP1, 4, and 6, show specificity for glutamates linked linearly on a side chain (Table 3; Rogowski et al., 2010; Wu et al., 2015, 2017). However, under the optimized condition, a biochemical assay demonstrated that CCP5 is able to cleave glutamates at both branched points and in linear side chains without the need for other CCP members (Table 3; Berezniuk et al., 2013).

**TABLE 3:** Members of the CCP family that act as deglutamylases.

Enzyme and Homologs	PTM	Substrate Preference	Subcellular/Ciliary Distribution	References
CCP1/Nan1 CCPP-1 (*Caenorhabditis elegans*)	Deglutamylation Δ2 modification Δ3 modification	Detyrosinated α-tubulin PolyE side chain Branching point E Non-Tubulin Protein(s): MLCK-1, Telokin, Klf4, 40S RPS9, TRAD1, HMGB1/2/3	Cilia Cell body (excluded from the nucleus) Dendrites §Also found in the nucleus of HeLa cells	Kalinina et al., 2007; Rogowski et al., 2010; O’Hagan et al., 2011; Berezniuk et al., 2012; De La Vega Otazo et al., 2013; Tort et al., 2014; Tanco et al., 2015; Bompard et al., 2018; Ye et al., 2018
CCP2	Deglutamylation Δ2 modification §No detyrosination or deglycylation	Detyrosinated α-tubulin Poly E side chain	Centrioles Basal bodies Cell body (excluded from the nucleus)	Kalinina et al., 2007; De La Vega Otazo et al., 2013; Tort et al., 2014
CCP3	Deglutamylation Δ2 modification Deaspartylation §No detyrosination or deglycylation	Detyrosinated α-tubulin Poly E side chain	Cell body (excluded from the nucleus)	Kalinina et al., 2007; Tort et al., 2014
CCP4	Deglutamylation Δ2 modification	Detyrosinated α-tubulin PolyE side chain Non-Tubulin Protein(s): MLCK-1, Telokin	Cell body (excluded from the nucleus)	Kalinina et al., 2007; Rogowski et al., 2010
CCP5/Agbl5 *CCPP-6 (*Caenorhabditis elegans*)	Deglutamylation	Branching point E Poly E side chain (short)	Cilia Cell body Nucleus Mitotic spindle microtubules Midbodies §Cell cycle–dependent distribution: in the nucleus during interphase; in mitotic spindle microtubules, midbodies during mitosis	Kalinina et al., 2007; Kimura et al., 2010; Rogowski et al., 2010; Ghosh-Roy et al., 2012; Berezniuk et al., 2013; De La Vega Otazo et al., 2013; Wu et al., 2017; He et al., 2018
CCP6	Deglutamylation Δ2 modification	Detyrosinated α-tubulin Poly E side chain (long) Non-Tubulin Protein(s): MLCK-1, Telokin, Klf4, Mad2	Cell body (excluded from the nucleus) Basal bodies Golgi apparatus Centrioles §Cell cycle–dependent distribution: in the Golgi apparatus, centrioles during interphase; in centrioles during mitosis	Kalinina et al., 2007; Rogowski et al., 2010; De La Vega Otazo et al., 2013; Ye et al., 2014, 2018

*There is no CCP5 in *C. elegans*, which do not possess motile cilia. CCPP-6 is functionally similar to CCP5 and is therefore taken to be the ortholog of CCP5.

### The Non-tubulin Substrates of Tubulin Modifying Enzymes

Apart from tubulins, both TTLLs and CCPs are able to modify substrates other than tubulins. TTLL1 as well as TTLL4 polyglutamate and stabilize the zinc finger transcription factor, Kruppel-like factor 4 (Klf4), by preventing its ubiquitination for further degradation, which therefore maintains the pluripotency of mouse embryonic stem cells (Ye et al., 2018). In pancreatic ductal adenocarcinoma cells, TTLL4 is also capable of chromatin remodeling for cell growth enhancement by glutamylating the transcription co-regulator, PELP1 (Proline, glutamic acid- and leucine-rich protein 1), and affecting its interaction with histone H3. As consequence, TTLL4 is considered as a candidate for pancreatic cancer treatment (Kashiwaya et al., 2010). For the cases of a histone chaperone, nucleosome assembly protein 1 (NAP1), TTLL4-dependent glutamylation enables its binding onto the plasma membrane of red blood cells (RBCs), which in turn affect the cell morphology. Though the details remain to be clarified, this suggests the role of TTLL4 in the organization of cytoskeletons in RBCs (Ijaz et al., 2017). *In vitro* assay for the enzyme activity of recombinant TTLL4 suggests its capability of glutamylating the recombinant murine NAP2 as well (Van Dijk et al., 2007). TTLL4 and TTLL6 act counter to CCP6 for polyglutamylation of Mitotic arrest deficient 2 (Mad2) in megakaryocytes (MKs). The polyglutamylated Mad2 is then able to promote the maturation of MKs and to regulate the subsequent production of platelets (Ye et al., 2014). Retinitis pigmentosa GTPase regulator (RPGR), a protein substrate of TTLL5, contains a basic domain that can specifically recruit TTLL5 to the cilia base and a Glu-Gly repetitive region that is architecturally similar to the C-terminal tail of α-tubulin. The glutamylation state of RPGR is relevant to the localization of cone opsins and the photoreceptor degeneration in mice (Sun et al., 2016).

Cytosolic carboxypeptidases have been taken as enzymes which hydrolyze the peptide bonds at the C-terminal of their substrates. In addition to tubulins, several proteins with C-terminal acidic tails have been predicted and verified as the substrate of CCP1, including telokin, Myosin light chain kinase (MLCK), ribosomal proteins (40S ribosomal protein S9), transcription factors (TRAF-type zinc finger domain-containing protein), and chromosomal proteins (high mobility group protein B1, B2, and B3). By modifying these proteins, CCP1 is capable of regulating various cell behaviors (Tanco et al., 2015). Moreover, CCP1 and CCP6 can modify Klf4 and counteract against TTLL1 and TTLL4 to stop HEK293T cells from reprogramming (Ye et al., 2018). A study in HEK293T cells suggested that CCP6 can also deglutamylate Telokin and MLCK as what CCP1 and CCP4 act (Rogowski et al., 2010).

## The Regulation of Cilia Architecture and Function by Axonemal Glutamylation

Based on the different lengths of the glutamic acid chains added to microtubules, glutamylation is able to fine-tune the regulation of diverse microtubule-based cell behaviors resulting from interactions with microtubule-dependent motors or -associated proteins (Verhey and Gaertig, 2007). Accumulating evidence highlight that loss of and excess glutamylation modification of the axoneme can both impact cilia architecture and/or function across ciliated species (Table 2). Here, we focus our discussion on how TTLL- and CCP-dependent glutamylation changes the stability and function of cilia and their involvement in signaling pathways and other physiological processes.

### The Role of Glutamylation in Primary Cilia Architecture

During zebrafish embryogenesis, both TTLL6-dependent glutamylation and CCP1/5-dependent deglutamylation are reported to be critical for ciliogenesis in olfactory placodes (Pathak et al., 2011; Lyons et al., 2013). In the CEM (Cephalic male) cilia of *C. elegans*, the cooperation between TTLL-11 and CCPP-1 remodels the axonemal doublets into a special formation of 18 singlets and then maintains them in this conformation (O’Hagan et al., 2017). Meanwhile, in the amphid neurons, TTLL4/5/11-dependent glutamylation of the axoneme counteracts with CCPP-1-mediated deglutamylation (Power et al., 2020). Hyperglutamylation of the axonemal tubulins due to TTLL4 overexpression or CCPP-1 deficiency may induce the spastin-dependent MT severing of the B tubules, which eventually leads to progressive defects in the ciliary structures and progressive degeneration as consequence (O’Hagan et al., 2011; O’Hagan and Barr, 2012). However, *in vitro* studies in HeLa cells have contrarily showed that the long side chains that result from TTLL6-dependent tubulin polyglutamylation regulate spastin-dependent microtubule-severing instead of the short ones generated by monoglutamylases such as TTLL4 or TTLL7 (Lacroix et al., 2010). These discrepancies might be explained by an *in vitro* study which found that glutamylation regulates spastin activity in a biphasic manner: There is a linear increase in the binding affinity of spastin to glutamylated microtubules and a non-linear decline in its severing activity. As a function of the polyglutamylation level and the side chain length, this may change the property of spastin from a severing enzyme to a stabilizer of microtubules, thus maintaining the architectural complexity of microtubule arrays (Valenstein et al., 2016).

### The Role of Glutamylation in Architecture and Motility of Motile Cilia

The proper level of glutamylation appears to be important for the structure and stability of axonemal microtubules in motile cilia as well. In *Chlamydomonas* flagella, polyglutamylation is mainly enriched on the microtubule cross-bridging N-DRC. The electrostatic interactions between negatively charged glutamic acids on B-tubule and the positive charges on DRC interlink the 9 + 2 conformation of *Chlamydomonas* axonemes (Kubo and Oda, 2017). The absence of TTLL5 in mice leads to the loss of doublet 4 and thus, disrupts axoneme 9 + 2 structure in sperm (Lee et al., 2013).

#### The Effects of Hypoglutamylation on Cilia

In sea urchin spermatozoa, injecting antibodies such as GT335 and B3 that can mask glutamylation, leads to defects in their beating amplitude but does not affect the flagellar beating frequency. In addition, microinjection of GT335 and B3 antibodies into human sperm or ciliated epithelial cells also impairs ciliary motility (Gagnon et al., 1996; Million et al., 1999; Ikegami and Setou, 2010). Deficiency in TTLL1 also leads to severe defects in the sperm flagella and the mid-pieces, which thereby disrupt the motility, and causes male infertility (Vogel et al., 2010). Despite the severe malformation in the sperm flagella in TTLL1 knockout mice, the architecture of the airway motile cilia are left intact. The loss of tubulin glutamylation causes the loss of the curvature and disrupts asymmetrical ciliary beating in trachea cilia, which can result in primary ciliary dyskinesia–like respiratory phenotypes, such as mucous accumulation or sneezing, in the individual (Ikegami et al., 2010). Genetic depletion of TTLL6 also suggests its unique role in regulating ciliary beating frequency in the ependymal cilia in mouse brains (Grau et al., 2013). In zebrafish, knockdown of TTLL3 and TTLL6 completely impairs the ciliary motility (Pathak et al., 2011). Deficiency of TTLL9 does not only cause reduction of glutamylation in doublet 5 but also shortening in the distal end of doublet 7. These disable the sperm from pro-hook bending and, therefore, halt the flagella beating of the mouse sperm (Konno et al., 2016). Tpg1, the TTLL9 homolog in *Chlamydomonas*, forms a complex with a flagella-associated protein FAP234 (Tpg2), which mainly functions in stabilizing tpg1 in the cytosol and in the subsequent IFT into the flagellar matrix. Defects in Tpg1 or Tpg2 lead to axonemal hypoglutamylation and thereby loss of the electrostatic interaction, which greatly decrease the flagellar motility without affecting the axoneme structure and dynein assembly (Kubo et al., 2010, 2012, 2014; Kubo and Oda, 2017).

#### The Effects of Hyperglutamylation on Cilia

In addition to the adverse impact of axonemal hypoglutamylation on ciliary motility, hyperglutamylation of the axonemes also affect ciliary motility. Hyperglutamylation on the B-tubule resulting from the overexpression of the TTLL6 homologs in *Tetrahymena thermophila* may hinders the microtubule sliding driven by inner dynein arms and thereby ciliary motility (Janke et al., 2005; Suryavanshi et al., 2010). CCP5 acts downstream of Fleer/IFT70 for tubulin deglutamylation, which halts ciliogenesis in zebrafish; Moreover, hyperglutamylation of axonemal microtubules in pronephric cilia induced by CCP5 deficiency also leads to motility defects and ciliopathy phenotypes (Pathak et al., 2014).

In summary, the proper level of glutamylation appears to be critical for generating proper motion in motile cilia or flagella across ciliated model organisms, which may be determined by whether erroneous axonemal architecture is present.

### The Roles of Glutamylation in IFT Dynamics

An *in vitro* study using chemically modified yeast tubulin with C-terminal glutamate side chains of various lengths showed a positive increase in both the progressivity and velocity of kinesin-2, the major motor in anterograde IFT (Sirajuddin et al., 2014). Mutation of CCPP-1 in *C. elegans* leads to abnormal accumulation of an anterograde motor, KLP-6 kinesin-3, and its cargo protein, polycystin-2, and to an increase in the rate of another anterograde motor OSM-3/KIF17 along the axoneme (O’Hagan et al., 2011). An *In vivo* study in *C. elegans* revealed that TTLL4 levels are affected by environmental stimuli and that the induced tubulin glutamylation also positively regulates kinesin-2–dependent IFT (Kimura et al., 2018). In addition, depletion of axonemal glutamylation preferentially hampers the anterograde IFT dynamics, with limited disruption of IFT dynamics in the opposite direction (Hong et al., 2018).

Two mechanisms might explain why axonemal glutamylation preferentially regulates anterograde IFT dynamics. (1) Evidence from correlative fluorescence and three-dimensional electron microscopy clearly demonstrates that anterograde IFT-B trains transport along the B-tubules, whereas retrograde IFT-A trains use the A-tubules as their railways (Stepanek and Pigino, 2016). Glutamylation appears to be more abundant on the B-tubules in various ciliated model organisms (Gagnon et al., 1996; Lechtreck and Geimer, 2000). Moreover, structural defects on B-tubules in cells with mutated CCPs and TTLLs have been frequently observed (Pathak et al., 2007, 2011; O’Hagan et al., 2012). Intuitively, defects on B-tubules caused by hypo/hyperglutamylation may disturb the railways of the IFT-B trains and therefore hamper anterograde IFT. Interestingly, evidence from cryo-electron tomography and subtomogram averaging reveals the appearance of the anterograde IFT trains along the A-tubule singlets in primary cilia of Madin-Darby Canine Kidney (MDCK) epithelial cells. This suggests different mechanisms of transport between the motile cilia and the primary cilia (Kiesel et al., 2020). Glutamylation statuses and the consequent effects on the A-tubule singlets in primary cilia still remain to be clarified. (2) *In vitro* studies demonstrated that glutamic acid chains enhance the processivity and velocity of kinesin-2 (Sirajuddin et al., 2014), and thus, hypoglutamylation of axonemes may slow down the kinesin-2–mediated anterograde IFT.

### The Roles of Glutamylation in Ciliary Signaling

As the proper cilia localization of many, if not all, signaling receptors/molecules depends on IFT transport, defects in IFT dynamics caused by hypo/hyperglutamylation would conceivably be expected to disturb ciliary signaling. Indeed, axonemal hypoglutamylation attenuates the translocation of GLi3 and tethering of Polycystic Kidney Disease 1/2 (PKD1/2) and affects ciliary Sonic Hedgehog (*Shh*) signaling and polycystin signaling, respectively (He et al., 2018). Consistently, axonemal deglutamylation using CCP5 deglutamylase artificially recruited onto axonemes also slows down the entry of Smoothened and Gli3 into the cilia, thereby blocking the corresponding *Shh* signaling (Hong et al., 2018). Since ciliary *Shh* signaling is required for the development and maintenance of various ciliated tissues (Bangs and Anderson, 2017), defects in Shh signaling induced by axonemal hyper/hypoglutamylation may cause systematically ciliopathy-relative phenotypes.

### Functional Crosstalk Between Tubulin Glutamylation and Glycylation

For various “tubulin code” that add along the axoneme via PTM modification, glutamylation and glycylation are special because, they may compete for the same glutamate on the C-terminus of tubulins (Pathak et al., 2011; Bosch Grau et al., 2017). Theoretically, in a biological compartment that possesses abundant modifying enzymes for two modifications that may compete for same sites, any changes leading to alteration of one modification will inevitably affect the occurrence of the other. Glycylation was first thought to be a modification enriched in motile cilia or flagella and plays a role in stabilizing the axoneme (Rogowski et al., 2009; Grau et al., 2013). In humans, the fact that TTLL10, the enzyme responsible for polyglycylation, is inactive indicates that polyglycylation *per se* is likely dispensable for ciliated cells (Rogowski et al., 2009). By using a specific monoclonal antibody that faithfully detects monoglycylation modification, axoneme monoglycylation could be detected in mouse neuronal cilia (Davenport et al., 2007), but not in many other types of primary cilia (Bré et al., 1996). This leads to the assumption that glycylation overall may be not essential for the structure and/or function of primary cilia. Interestingly, recent evidence demonstrated that primary cilia, at least in some cell types, may depend on monoglycylation to maintain proper structure and function (Rocha et al., 2014; Bosch Grau et al., 2017; Gadadhar et al., 2017b). TTLL3 is an enzyme that catalyzes tubulin monoglycylation in the axoneme (Wloga et al., 2009). TTLL3 deficiency in either *Tetrahymena* or zebrafish results in shortened axonemes, which is thought to act either directly or indirectly by altered tubulin glutamylation (Wloga et al., 2009). In *Ttll3* knockout mice, reduced tubulin monoglycylation leads to an increased level of tubulin glutamylation in photoreceptor cells, and contributes to shortened connecting cilia and retinal degeneration (Bosch Grau et al., 2017). Coincidently, *pcd* mice which carry a Ccp1-inactivating mutation also show progressive degeneration of photoreceptors (Bosch Grau et al., 2017). To this end, it is worth investigating the exact contribution of hypoglycylation or hyperglutamylation of connecting cilia to retinal degeneration. What adds another level of complexity is that axoneme glycylation and glutamylation also show overlapping roles in maintenance of cilia structure and motility in zebrafish (Pathak et al., 2011). Taken together, while analyzing phenotypes caused by alterations of either axoneme glycylation (specifically monoglycylation for human cells) or glutamylation, it needs to be kept in mind that hyperglycosylated axoneme is very likely accompanied by less axoneme glutamylation or *vice versa*, especially in motile cilia or flagella. Future identification of ciliary effectors/mechanisms recognizing (poly)glutamylation and (poly)glycylation modifications will guarantee a thorough understanding of the underlying crosstalk between these two modifications on the C-terminal tail of axonemal tubulins.

## Dysregulation of Axoneme Glutamylation and Human Ciliopathies

### Hypoglutamylation and Joubert Syndrome

Although studies in mice suggest that hypoglutamylation is correlated with several classical ciliopathy phenotypes associated with dysfunction of motile cilia, such as respiratory disorders (Ikegami et al., 2010), dysfunctional ependymal cilia in the brain ventricles (Grau et al., 2013), and infertility (Campbell et al., 2002; Ikegami et al., 2007; Lee et al., 2013; Konno et al., 2016), its physiological importance in human health has historically been overlooked. Extensive research on cilia biology, in the last 20 years – especially the rapid cloning and characterization of causative genes underlying ciliopathies – have begun to uncover the critical role of axoneme glutamylation in the pathogenesis of human diseases.

Joubert syndrome is a genetically heterogeneous group of disorders characterized by a malformed brain stem (molar tooth sign), and is accompanied by other non-central nervous system-related ciliopathy phenotypes including retinal degeneration, polydactyly, and renal/liver abnormalities (Saraiva and Baraitser, 1992; Cantagrel et al., 2008). The first evidence linking dysregulation of axoneme glutamylation to human ciliopathies was the discovery that causative mutations in the centrosomal protein CEP41, which is mutated in Joubert syndrome, does not affect cilia biogenesis but disrupts the proper ciliary entry of the glutamylase TTLL6, which leads to dramatically reduced polyglutamylation along the axoneme in primary cultured fibroblasts isolated from Joubert syndrome patients (Lee et al., 2012). Depletion of CEP41 in zebrafish and mice causes similar defects in axoneme glutamylation and ciliopathy-related phenotypes (Lee et al., 2012). Shortly after the characterization of CEP41 as a Joubert syndrome protein, homozygous mutations in TTLL5 were reported to cause retinal dystrophy in a subset of patient families with inherited retinal degenerations (Sergouniotis et al., 2014).

To date, 37 Joubert syndrome genes have been cloned, although the functions of most encoded proteins remain elusive. The perspective that dysregulated axoneme glutamylation might be a central etiology in JBTS was further strengthened by the finding that the Joubert syndrome protein ARL13B associates with FIP5, a known effector of another ciliary GTPase RAB11, to promote the ciliary import of tubulin glutamylases TTLL5 and TTLL6 in human epithelial cells (He et al., 2018). A defective ARL13B-FIP5 pathway leads to axoneme hypoglutamylation, which does not affect ciliogenesis but does promote the disassembly of cilia and, importantly, impairs polycystin and shh signaling by disrupting the proper trafficking of various signaling molecules in the cilia (He et al., 2018). Amazingly, restoring axoneme glutamylation by depleting the cilia-enriched deglutamylase CCP5 can effectively rescue ciliary defects in *ARL13B*-deficient cells (He et al., 2018).

An intriguing discovery is the very recent finding that a TOG array regulator of axonemal microtubules 1 (TOGARAM1)-Armadillo repeat containing 9 (ARMC9) module may regulate both axoneme acetylation and polyglutamylation in human and zebrafish (Latour et al., 2020). *TOGARAM1* and *ARMC9* were separately identified as causal loci of Joubert syndrome (Van DeWeghe et al., 2017; Latour et al., 2020; Morbidoni et al., 2020). It is not known why and how the TOGARAM1-ARMC9 module regulates both acetylation and glutamylation of the axoneme. It is also not yet conclusive whether defective acetylation or defective glutamylation of the axoneme contributes to the ciliopathy phenotypes associated with *TOGARAM1* and *ARMC9* patients. However, as defective axoneme acetylation is not observed in *CEP41*- or *ARL13B*-deficient cells (Lee et al., 2012; He et al., 2018), we reason that defective axoneme glutamylation is probably the major driver for the development of ciliopathy phenotypes observed in Joubert syndrome patients.

### The Effect of Axoneme Hyperglutamylation on Human Health

Tubulin polyglutamylation is enriched during neuronal differentiation and is therefore, considered as a potential key physiological regulator of neuronal cells. Microtubule hyperglutamylation of neuronal axons is also associated with neural degeneration in humans and in *Ccp1*^–/–^ mice (Rogowski et al., 2010; Shashi et al., 2018). In *pcd* mice (carrying a *Ccp1*-inactivating mutation), the Purkinje cell degeneration phenotype directly links tubulin hyperglutamylation to neurodegeneration (Mullen et al., 1976; Greer and Shepherd, 1982; Fernandez-gonzalez et al., 2002; Rogowski et al., 2010; Shashi et al., 2018). Although no mutations of known ciliopathy proteins have been reported to cause hyperglutamylation of the axoneme in human so far, given that polyglutamylation is also highly enriched in the axoneme, it is thus, a natural question to ask whether axoneme hyperglutamylation has any adverse impact on human cilia and, consequently, can be detrimental to human health. As we discussed in section “The Effects of Hyperglutamylation on Cilia,” tubulin hyperglutamylation adversely affects cilia in some ciliated species, especially that tubulin hyperglutamylation is implicated in regulating microtubule severing or the motility of flagella or motile cilia (Roll-Mecak and Vale, 2008; Lacroix et al., 2010; Grau et al., 2013; Valenstein et al., 2016). Hyperglutamylation in *Ccp1*^–/–^ (Mullen et al., 1976; Bosch Grau et al., 2017) or *Ccp5*^–/–^ mice (Wu et al., 2017; Giordano et al., 2019) also share phenotypes including infertility and abnormal sperm biogenesis. However, hyperglutamylation appears to be benign to primary cilia in mammalian cells. In cultured human or mouse cells, axoneme hyperglutamylation induced by depleting the cilia-enriched deglutamylase CCP5 produces significantly longer cilia and enhances ciliary signaling by promoting the import of signaling molecules but does not result in detectable ciliary anomalies in primary cilia (He et al., 2018). Consistently, for the three deglutamylases (CCP2, 3, 5) that are reported to be ciliary deglutamylases, *Ccp2*^–/–^ and *Ccp3*^–/–^ (Tort et al., 2014), and *Ccp5*^–/–^ (Xia et al., 2016; Wu et al., 2017) mice are viable and generally healthy without classical phenotypes associated with dysfunctional primary cilia.

Except for aforementioned sperm-related phenotypes, hyperglutamylation has been correlated with retinal degenerations in humans (Sergouniotis et al., 2014; Kastner et al., 2015; Astuti et al., 2016; Branham et al., 2016) and in mouse models (Marchena et al., 2011; Bosch Grau et al., 2017). Intriguingly, it remains unknown whether hyperglutamylation-associated retinal degeneration is caused by alteration of polyglutamylation levels along the axoneme or it is a tubulin-associated defect. Evidence suggests that TTLL and CCP enzymes involved in glutamylation modification can also target many non-tubulin substrates (for detailed discussion, please see section “The Non-tubulin Substrates of Tubulin Modifying Enzymes”). Interestingly, in mice, the RPGRORF15 (the photoreceptor-specific ORF15 variant of retinitis pigmentosa GTPase regulator) implicated in retinal dystrophy actually localizes to connecting cilia of photoreceptors and is glutamylated by TTLL5 *in vivo* (Rao et al., 2016). Other studies show that TTLL5 deficiency also disrupts the glutamylation of RPGR and leads to retinal pathology, without detectable changes in the level of tubulin glutamylation and axonemal structure of connecting cilia (Lee et al., 2013; Sergouniotis et al., 2014; Sun et al., 2016). Thus, it should be cautious when drawing conclusions that link tubulin glutamylation to the *in vivo* phenotypes observed in conditions with altered TTLL or CCP enzyme activities.

Collectively, this evidence suggests that the impact of axonemal hyperglutamylation appears to be benign, at least, in the context of primary cilia. This is important because hyperglutamylation induced by CCP5 depletion, can effectively restore axonemal glutamylation, the ciliary dosage of polycystins, and *Shh* signaling in human wild-type or Autosomal dominant polycystic kidney disease (ADPKD) cells (He et al., 2018), which highlights the perspective that augmenting ciliary signaling by increasing axoneme glutamylation may represent an intriguing therapeutic strategy for certain ciliopathies.

## New Methods Enabling the Spatiotemporal Manipulation of Axonemal Glutamylation

Typically, three strategies are used to study tubulin glutamylation: (1) genetic perturbation of genes that encode glutamylation-modifying enzymes, (2) manipulation of the glutamylation levels of purified microtubules by recombinant enzymes *in vitro*, and (3) introduction of specific antibodies to mask the glutamylated motifs of microtubules. Although all of the above experiments imply that glutamylation is important for the structural integrity and functions of cilia (Gaertig and Wloga, 2008; Konno et al., 2012; Grau et al., 2013; Pathak et al., 2014; Chawla et al., 2016), these conventional methods have several limitations.

First, tubulin glutamylation is not restricted only to ciliary axonemes in cells. Glutamylated tubulins are also enriched at centrosomes, mitotic spindles, and intercellular bridges (Wloga et al., 2010; Janke and Bulinski, 2011), perhaps owing to the distribution of TTLLs and CCPs among multiple subcellular compartments (Tables 1, 3; Van Dijk et al., 2007; He et al., 2018). Therefore, global perturbation of modifying enzymes by conventional gene manipulation does not affect glutamylation only in primary cilia. The effects of hypo/hyperglutamylation in genetically modified cells are in fact, the combined outcome resulting from, at a minimum, the perturbed microtubule pools at each of the sites where the modifying enzyme is located. The introduction of antibodies against glutamylated tubulin into cells would suffer from the same limitation, as it globally masks all glutamylated tubulin pools in cells.

Second, the interplay among microtubules, motor proteins, and other MAPs is highly dynamic. For example, tubulin glutamylation occurs on the surface of microtubules and regulates IFT dynamics while the IFT cargo, such as modifying enzymes, microtubule precursors, and other MAPs, also dynamically regulate the PTM of microtubules, as well as their structure and functions (O’Hagan et al., 2017). Thus, long-term gene manipulation does not allow for a time window to dissect these dynamic processes and also presents challenges with respect to uncovering causal relationships.

Third, *in vitro* modification of purified microtubules by recombinant enzymes enables the study of acute effects of PTMs on the physical properties of microtubules and their interactions with motor proteins and MAPs. However, it is difficult to fully reconstitute the physiological environment and to include all relevant cellular molecules in *in vitro* systems.

Last, but not least, as discussed above (section “The Non-tubulin Substrates of Tubulin Modifying Enzymes”), tubulins are not the only substrate for glutamylation modification. Many nucleocytoplasmic shuttling proteins such as the nucleosome-assembly proteins NAP1 and NAP2 are also identified as potential substrates of TTLL4-mediated glutamylation (Regnard, 2000; Ijaz et al., 2017). The phenotypes triggered by global perturbation of TTLLs and CCPs are combinational outcomes of both tubulin and non-tubulin substrate events. Therefore, owing to the above reasons, these conventional methods are not feasible to study the specifical role of axonemal glutamylation.

**FIGURE 4:**
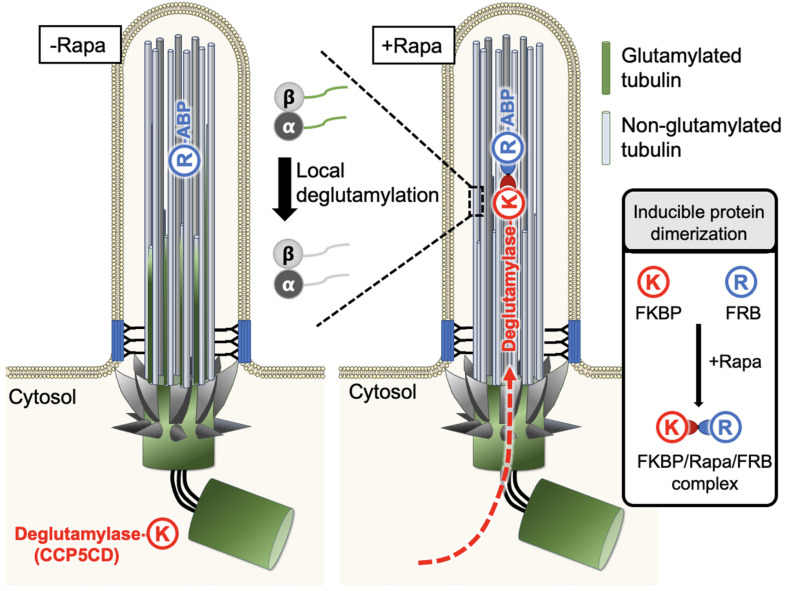
A new approach enables spatiotemporal manipulation of tubulin PTMs in living cells. Typically, FRB (R) can be anchored to the region of interest with the help of a specific targeting motif, whereas FKBP (K) is fused to the protein of interest, which is functional only upon recruitment to the FRB-labeled sites and stays non-functional otherwise. This characteristic feature was used to manipulate glutamylation on ciliary axonemes in a rapamycin-mediated IPD manner. FRB was tagged with an axonemal binding protein (ABP), MAP4m (a truncated mutant of microtubule-associated protein 4), and the resultant fusion protein was anchored on the ciliary axonemes. The second fusion protein was constructed by fusion of the catalytic domain of CCP5 deglutamylase (CCP5CD) with FKBP, this soluble protein can freely diffuse in the cytosol without noticeable deglutamylation activity due to its low microtubule affinity. As the cytosolic proteins can freely access the primary ciliary lumen, the addition of rapamycin traps CCP5CD-FKBP fusion protein on the FRB-tagged axonemes. Once stationed at FRB-tagged sites, CCP5CD depletes glutamylated tubulin on ciliary axonemes locally and efficiently without affecting other glutamylated microtubules in the cells.

Explicitly studying the impact of glutamylation modification on cilia architecture and functions, an emerging new approach that spatiotemporally recruits PTM-modifying enzymes to specific pools of microtubules on the basis of inducible protein dimerization (IPD) may address this long-standing issue (Hong et al., 2018). The IPD system usually involves three components, a chemical cross-linker and a pair of binding partners (Banaszynski et al., 2005). One well-established IPD system uses a natural chemical cross-linker, rapamycin, to induce the dimerization between two soluble proteins, FKBP (FK506 binding protein) and FRB (FKBP-rapamycin binding domain) (Banaszynski et al., 2005). Typically, FRB is anchored to the region of interest by tagging it with a specific targeting motif, whereas FKBP is fused to a protein of interest that is non-functional when freely dissociated within the cytosol but is activated upon recruitment to the FRB-labeled sites (Figure 4). The addition of rapamycin brings the FKBP fusion protein to the FRB-residual site where the FKBP-tagged protein of interest can access its substrates or downstream signaling molecules to locally manipulate the corresponding molecular activities. Thus far, the IPD system has been extensively used to manipulate the activity of small GTPases, lipid homeostasis, cellular mechanotransduction, and so on (Varnai et al., 2006; Zoncu et al., 2007; Bohdanowicz and Fairn, 2011; DeRose et al., 2012; Fan et al., 2017; Wang Y. et al., 2019).

Taking advantage of the above characteristics, Hong et al. (2018) recently used rapamycin-mediated IPD to spatiotemporally manipulate tubulin glutamylation on ciliary axonemes. In this system, FRB was tagged with an axoneme binding protein, MAP4m (a truncated mutant of MAP 4), and the resulting fusion protein was consistently localized to the ciliary axoneme. On the other side, the catalytic domain of CCP5 deglutamylase (CCP5CD) was fused with FKBP, and this soluble fusion protein freely diffused in the cytosol without noticeable deglutamylation activity, perhaps owing to its low affinity for microtubules. As the cytosolic proteins can freely access the lumen of primary cilia (Lin et al., 2013), the addition of rapamycin rapidly trapped the CCP5CD-FKBP fusion protein on axonemes where CCP5CD locally and efficiently depleted glutamylated tubulin on ciliary axonemes with no or rare adverse effects on other glutamylated microtubule pools in cells. The direct effects of deglutamylation can, therefore be validated by the comparison of ciliary structures and functions before and after acute axonemal deglutamylation. The results demonstrate that depletion of glutamylation on ciliary axonemes reduces the rate of ciliogenesis but does not perturb ciliary maintenance. In addition, axonemal deglutamylation attenuates anterograde IFT and suppresses ciliary Shh signaling. An advantage of this approach is that it enables the spatiotemporal manipulation of tubulin glutamylation in living cells and thus, can uncover specific roles of glutamylation in certain subcellular areas. Different microtubule-binding proteins and enzymes responsible for distinct tubulin PTMs can be engineered and applied in this system to precisely modify distinct tubulin PTMs in specific subcellular regions.

One drawback of chemically based IPD is poor reversibility. The protein complex triggered by chemical dimerizers can not be easily split and, therefore, the manipulated system generally can not be halted (DeRose et al., 2013). Moreover, it is not easy to provide spatial precision of manipulation by chemically based IPD due to free diffusion of dimerizers. To improve these limitations, optogenetic systems that use light as an external cue to control the behavior of microtubules have been developed. These systems are achieved by blue light–triggered dimerization of different partners including iLID/SSPB (Adikes et al., 2018), Cry2/CIBN (Prosseda et al., 2020), and LOV2/Zdk1 (VanHaren et al., 2018; vanHaren et al., 2020). The crosslink between the microtubule plus end and F-actin that is triggered by the light temporally perturbs the microtubule dynamics in living cells (Adikes et al., 2018). Microtubule gliding activity can also be controlled by light-inducible recruitment of motor proteins to the microtubule surface (Tas et al., 2018). In the studies from VanHaren et al. (2018) and vanHaren et al. (2020), they designed a light-inactivated π-EB1 that can attenuate microtubule growth under blue light illumination (VanHaren et al., 2018; vanHaren et al., 2020).

Along with studying microtubule dynamics, optogenetic systems have recently been used to study cilia and flagella. The cAMP level in primary cilia or flagella can be regulated by light-activated phosphodiesterase (LAPD), which therefore allows scientists to temporally manipulate sperm motility or to study the spatial aspect of cAMP signaling in primary cilia (Raju et al., 2019; Hansen et al., 2020). In addition, with the Cry2/CIBN-based optogenetic method, scientists can spatiotemporally manipulate the level of phosphoinositide in primary cilia, which regulates eye pressure (Prosseda et al., 2020). These optogenetic tools should also have applications with respect to spatiotemporally and reversibly manipulate tubulin PTMs under the control of light illumination. However, the proper modifying enzyme that can rapidly and efficiently perturb tubulin PTMs is highly desirable for minimizing the phototoxicity from long-term light illumination.

## Conclusion and Perspective

Evolution has shown that glutamylation is functionally vital for the cells that have retained cilia or flagella. Indeed, *in vivo* deficiency of enzymes or regulatory proteins required for axonemal glutamylation causes erroneous pattern and level of tubulin glutamylation in cilia, which is accompanied by the impairment of cilia architecture and/or functions ranging from cilia formation, IFT dynamics, motility, to signaling. However, owing to the non-specific enzymatic activities and poor spatiotemporal accuracy of PTM perturbation with the current methods, caution is needed with respect to the interpretation of the roles of PTMs in cellular functions. Increasing structural and biochemical evidence has shown the detailed mechanisms by which tubulin PTM–modifying enzymes interact with microtubules and execute their enzymatic reactions (Mukai et al., 2009; Otero et al., 2012; Tort et al., 2014; Garnham et al., 2015, 2017; Wu et al., 2015; Miyake et al., 2016; Park et al., 2016; Natarajan et al., 2017; Skultetyova et al., 2017; Adamopoulos et al., 2019; Eshun-Wilson et al., 2019; Li et al., 2019, 2020; Liao et al., 2019; Liu et al., 2019; Wang N. et al., 2019; Zhou et al., 2019; Janke and Magiera, 2020; Mahalingan et al., 2020; Ustinova et al., 2020). These studies provide important fundamental information for engineering tubulin PTM–modifying enzymes with better substrate specificities and vigorous enzyme activity. This information, together with emerging approaches that enable spatiotemporal recruitment of engineered enzymes onto specific microtubule populations, pharmacological discovery of small molecules that can specifically regulate glutamylase/deglutamylase enzymatic activities, and identification of specific effectors/adaptors that recognize tubulin PTMs, should help to uncover the specific roles of axonemal PTMs in ciliated cells and elucidate the physiological importance in human health. In addition to insights into how tubulin PTMs regulate cellular architecture and activities, such approaches/compounds that can manipulate tubulin PTMs may offer a fundamental step toward facilitating the development of therapeutic strategies for tubulin PTM–related diseases.

Additionally, the reagents and sensitive methods allowing for monitoring the properties and behavior of microtubule population are highly desirable for the progress of this field. For instance, the detection of tubulin PTMs in cells is mainly based on the immunostaining with specific antibodies which is challenging to apply in live-cell experiments. Development of biosensors which can label certain microtubule populations that carry specific PTMs with limited adverse effects can be utilized to monitor the real-time behaviors of microtubules with corresponding PTMs in living cells under different physiological and pathological conditions. Very recently, Kesarwani et al. (2020) established a genetically encoded biosensor that specifically recognizes tyrosinated microtubules *in vitro* and *in vivo*, thus offering a feasible tool to visualize and quantify the level of tyrosinated microtubules in a real-time manner. Development of these reagents and tools will broaden our understanding of how tubulin PTMs regulate cells in space and time.

## Author Contributions

All authors wrote the manuscript, tables, and drew the figures.

## Conflict of Interest

The authors declare that the research was conducted in the absence of any commercial or financial relationships that could be construed as a potential conflict of interest.
